# Spatial structuring of the population genetics of a European subterranean termite species

**DOI:** 10.1002/ece3.1566

**Published:** 2015-07-08

**Authors:** Stéphanie Bankhead-Dronnet, Elfie Perdereau, Magdalena Kutnik, Simon Dupont, Anne-Geneviève Bagnères

**Affiliations:** 1 Laboratoire de Biologie des Ligneux et des Grandes Cultures, EA 1207, Université d’Orléans45067, Orléans, France; 2 Institut de Recherche sur la Biologie de l’Insecte, UMR 7261 CNRS - Université François Rabelais. UFR Sci. & Tech.Tours, 37200, France; 3 FCBA - Institut technologique, Dpt Biologie et Préservation du BoisAllée de Boutaut BP227, 33028, Bordeaux, France

**Keywords:** Bayesian clustering, breeding system, population structure, *Reticulitermes grassei*, spatial genetics, spatial principal component analysis

## Abstract

In population genetics studies, detecting and quantifying the distribution of genetic variation can help elucidate ecological and evolutionary processes. In social insects, the distribution of population-level genetic variability is generally linked to colony-level genetic structure. It is thus especially crucial to conduct complementary analyses on such organisms to examine how spatial and social constraints interact to shape patterns of intraspecific diversity. In this study, we sequenced the mitochondrial COII gene for 52 colonies of the subterranean termite *Reticulitermes grassei* (Isoptera: Rhinotermitidae), sampled from a population in southwestern France. Three haplotypes were detected, one of which was found exclusively in the southern part of the study area (near the Pyrenees). After genotyping 6 microsatellite loci for 512 individual termites, we detected a significant degree of isolation by distance among individuals over the entire range; however, the cline of genetic differentiation was not continuous, suggesting the existence of differentiated populations. A spatial principal component analysis based on allele frequency data revealed significant spatial autocorrelation among genotypes: the northern and southern groups were strongly differentiated. This finding was corroborated by clustering analyses; depending on the randomized data set, two or three clusters, exhibiting significant degrees of differentiation, were identified. An examination of colony breeding systems showed that colonies containing related neotenic reproductives were prevalent, suggesting that inbreeding may contribute to the high level of homozygosity observed and thus enhance genetic contrasts among colonies. We discuss the effect of evolutionary and environmental factors as well as reproductive and dispersal modes on population genetic structure.

## Introduction

Subdivisions within populations can provide valuable insights when deciphering the ecological and evolutionary processes operating in natural populations. In most species, population structure is affected by habitat characteristics, life history traits, and nonrandom mating among individuals (Barton and Clark 1990). Population genetics studies that focus on social insects may be of particular interest; the colony is an additional level that must be considered in hierarchically structured populations (Vargo 2003). For example, social insects are attracting increased attention as a study system because they reveal how breeding and dispersal affect the distribution of genetic variation (Ross and Keller 1995).

Population genetics studies of *Reticulitermes* subterranean termite species (Isoptera, Rhinotermitidae) have been multiplying over the past fifteen years. Small-scale genetics studies have focused on characterizing colony breeding systems and delineating colony foraging areas, in both forests and urban areas; polymorphic microsatellite markers have been used to examine how genetic variation is partitioned within and among colonies (e.g. *Reticulitermes flavipes,* Vargo 2003; Vargo and Carlson 2006; *R. hageni,* Vargo and Carlson 2006; *R. virginicus*, and *R. hageni*, Vargo et al. 2006; *R. grassei,* DeHeer et al. 2005; Nobre et al. 2008; *R. chinensis*, Huang et al. 2013). Considerable variation in colony breeding systems and the proportions of different family structures within and among species have been found (*e.g*., in the number of reproductives and their level of relatedness within colonies; reviewed in Vargo and Husseneder 2009, 2011). In the new ranges of some introduced populations, colony genetic structure may exhibit very particular patterns, such as the presence of hundreds of inbred neotenic reproductives and increased colony fusion (*R. flavipes* [=*R. santonensis*], introduced from the USA to France and other countries (Dronnet et al. 2005, 2006; Perdereau et al. 2010, 2013b, 2015); and *R. urbis*, introduced from the Balkans to France and Italy (Leniaud et al. 2010; Perdereau et al. 2013a). Therefore, investigating the population genetics of *Reticulitermes* species, whether endemic or introduced, may yield information that clarifies how breeding structure influences large-scale genetic differentiation (i.e. among populations). Indeed, colony breeding structure and modes of dispersal (natural flight, subterranean budding, or human-mediated movement) act to shape complex termite populations that are also undergoing drift and selection. In particular, they can affect population inbreeding, which, in turn, can influence the degree of genetic differentiation among populations (reviewed in Vargo and Husseneder 2009, 2011).

The European subterranean termite *Reticulitermes grassei* is an endemic termite species that is naturally found throughout Spain, Portugal, and southwestern France. Three past studies have used phylogeographic analyses to determine how historical events explain the current geographical distribution of *R. grassei* populations; researchers examined populations on the Iberian Peninsula and in southwestern France (Kutnik et al. 2004; Lefebvre T., Kutnik M., Zimmermann M., Dupont S., Vargo E.L. and Bagnères A.-G., submitted), as well as colonies occurring along a 640-km north–south transect in Portugal (Nobre et al. 2006). However, studies of historical gene flow do not necessarily reveal contemporaneous gene flow, particularly that which occurs in human-shaped landscapes or in response to long-standing geographical barriers, such as the Pyrenees. Other genetic studies of *R. grassei* have examined the colony breeding systems of one population occurring on a plantation in Portugal (Nobre et al. 2008), as well as those of four populations found in forests in southwestern France (DeHeer et al. 2005; Perdereau et al. 2011). Information on the breeding systems and inbreeding levels of three of these four *R. grassei* French populations, along with data from three Spanish populations, suggest that a strong, positive relationship exists between latitude and inbreeding in *R. grassei* colonies: The species prefers cool and moist conditions (Vargo et al. 2013). However, the degree to which regional divergence among populations is related to levels of inbreeding and/or modes of dispersal needs to be explored further.

The objectives of this study were to explore how a complementary analytical approach, employing frequency- and model-based methodologies, could disentangle spatial patterns of genetic differentiation among 52 colonies of the endemic termite species *R. grassei* found in southwestern France. These analyses were carried out with and without taking into account the geographic dimension of the genetic data by explicitly integrating or excluding spatial information. This is the first study to date to exploit spatial framework tools and a combination of conventional genetic statistics, multivariate analyses, and Bayesian approaches to examine termite population genetic structure at a regional scale. We found that such analytical methods may be particularly helpful in social insect studies that seek to explain the distribution of population-level genetic variability, which is linked to colony-level genetic structure.

## Materials and Methods

### Sample collection and DNA extraction

**Figure 1:**
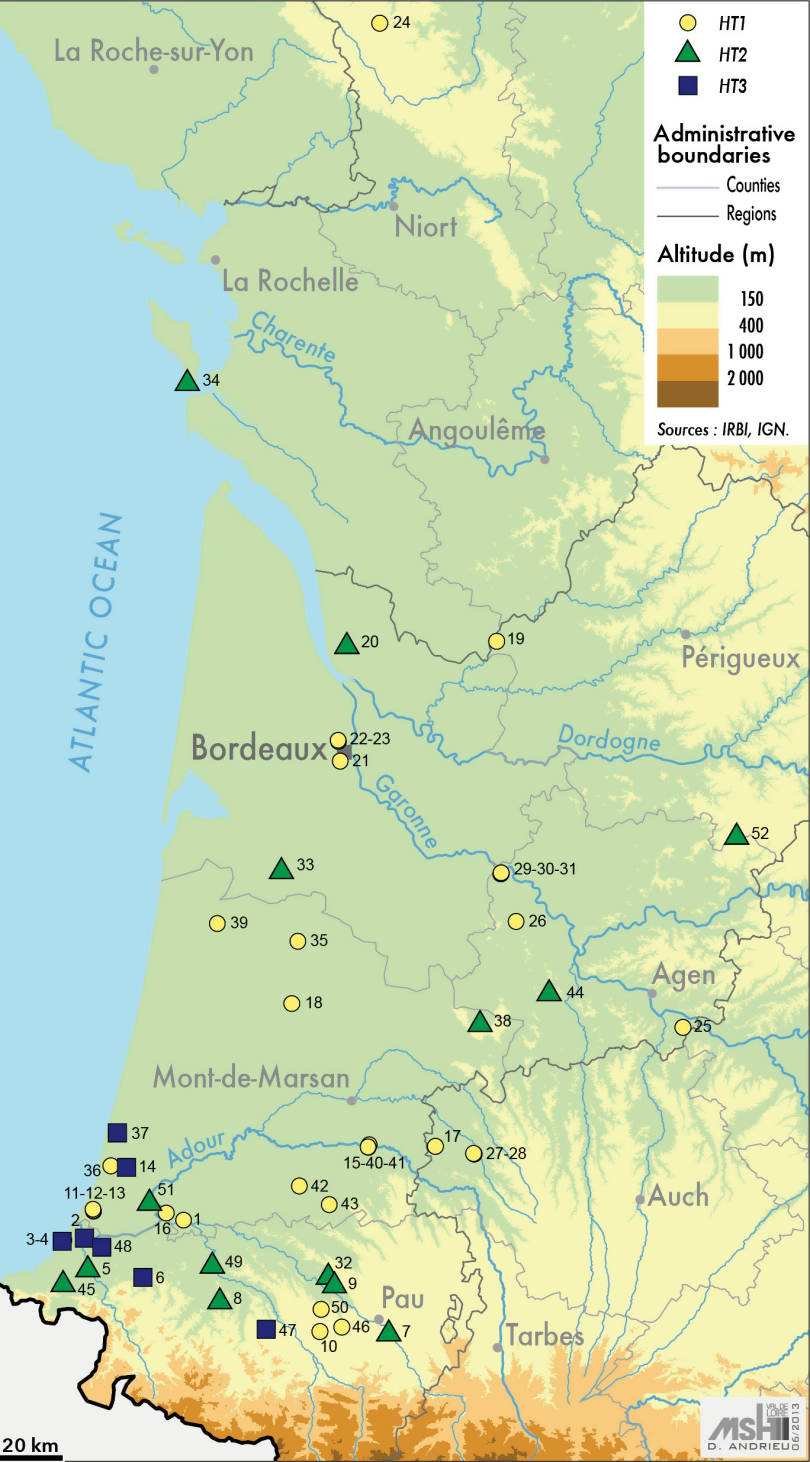
Spatial distribution of the 52 *Reticulitermes grassei* colonies sampled in southwestern France. The color of the symbols indicates the specific COII mtDNA haplotype identified using one individual per colony: HT1 (yellow circle), HT2 (green triangle), and HT3 (blue square).

Samples of *R. grassei* were collected in both forests and urban areas in southwestern France between June 2002 and April 2004; the sampling area spanned 200 km from east to west and 395 km from north to south. We collected ten workers from each of the 52 colonies sampled (Fig.1 and Table S1). In forests, termites were found feeding upon stumps or felled trees. All termites were placed in 99% ethanol and stored at room temperature (20°C) until DNA extraction and genetic analyses could take place. Genomic DNA was extracted from whole termite bodies using the method described in Kutnik et al. (2004).

### PCR amplification and sequencing of the mtDNA region

We screened for mitochondrial DNA variation by sequencing a 684-bp fragment of the cytochrome oxidase subunit II (COII) gene for one individual from each of the 52 colonies using the primers TL2-J-3037 (5′– ATG GCA GAT TAG TGC AAT GG –3′) and TK-N-3785 (5′–GTT TAA GAG ACC AGT ACT TG–3′) (Austin et al. 2002). The amplification procedure consisted of an initial 5-min melting step at 94°C, denaturation, 35 iterations of the temperature cycle (94°C for 1 min, 48°C for 1 min, and 70°C for 1 min), and a 7-min final extension step at 70°C. PCR products were purified using a Qiagen PCR Purification Kit (Qiagen, Hilden, Germany) and directly sequenced using a capillary DNA sequencer (ABI PRISM 3100). COII sequences were aligned using the ClustalW algorithm in the BioEdit 6.0.7 sequence alignment editor (Hall 1999) and then corrected manually.

### Microsatellite genotyping

We genotyped 512 termite workers from 52 colonies at 6 microsatellite loci: *Rf6-1*, *Rf5-10*, *Rf21-1*, *Rf24-2*, *Rf15-2* (Vargo 2000), and *Rs76* (Dronnet et al. 2004). The amplification of all the primers was performed using a Robocycler® Gradient 96 (Stratagene) and the PCR conditions described in Dronnet et al. (2004), with the exception of two modifications. The amplification of the primers for *Rf15-2* and *Rs76* was performed using a single reaction, in which final primer concentrations were 50 *μ*mol/L for *Rf15-2* and 30 *μ*mol/L for *Rs76* and the annealing temperature was fixed at 57°C. The remaining loci were amplified using two multiplexed reactions (*Rf24-2* with *Rf6-1* and *Rf21-1* with *Rf5-10*). The final primer concentrations were 30 *μ*mol/L for *Rf21-1* and *Rf24-2* and 100 *μ*mol/L for *Rf5-10* and *Rf6-1*. PCR products were separated by electrophoresis on 6% polyacrylamide gels run on a LI-COR 4000 L sequencer, and allele sizes were determined by comparison with a 50- to 350-bp IRDye 800™ standard (Li-Cor Inc., Lincoln, NE, USA). Alleles were scored using the program GeneProfiler, v. 4.03 (Scanalytics, Inc., Fairfax, VA, USA).

### Genetic data analyses

#### Standard population genetic analyses of the full sample

First, we determined the number of polymorphic nucleotide sites and haplotypes for the COII fragment using BioEdit 6.0.7 (Hall 1999). Second, the number of alleles per locus, observed heterozygosity *H*_O_, and Nei’s unbiased expected heterozygosity *H*_E_ (Nei 1987) at the six microsatellite loci were calculated using fstat v. 2.9.3 (Goudet 2001). We tested for deviations from Hardy–Weinberg equilibrium (HWE) at each locus and overall using the exact tests of heterozygote deficiency in genepop (online version 4.2; Raymond and Rousset 1995). In order to quantify such deviations, we calculated Weir & Cockerham’s estimate of *F*_IS_ (1984) for each locus and overall. We tested for genotypic linkage disequilibrium for each pair of loci, applying the Bonferroni correction for multiple comparisons. To prevent within-colony relatedness from skewing the results, we used the following approach (see Vargo 2003). A single individual was chosen from each colony using a random number table. The data for these individuals were combined to construct a data set. Each colony was resampled 20 times total to generate 20 replicated data sets.

An analysis of molecular variance (AMOVA, implemented in arlequin v. 3.11; Excoffier et al. 2005) was used to partition the total molecular variance into different hierarchical levels (i.e. among colonies, among individuals within colonies, and within individuals).

We tested for the presence of an isolation-by-distance (IBD) pattern between colonies (Slatkin 1993; Rousset 1997). Using the replicated data sets described above, we tested the correlation between Edwards’ distances and Euclidean geographic distances for pairs of colonies (Cavalli-Sforza and Edwards 1967; Edwards 1971) using a Mantel test (999 permutations; Mantel 1967) implemented with the adegenet v. 1.4.2 and ade4 v. 1.6-2 packages in R v. 3.0.3. A classical IBD pattern should result in a continuous cline of genetic differentiation and a correlation between the two distance parameters. However, distant and differentiated populations would also exhibit such a pattern. To disentangle these different possible scenarios, we visually assessed local point density using a colored scatterplot (two-dimensional kernel density estimation, *kde2d* function in the mass v. 7.3-29 package and the adegenet v. 1.4.2 package), which showed either a single consistent cloud of points (continuous cline) or several patches (distant populations) (Jombart 2008).

### Genetic structure revealed by multivariate analyses

To explore and detect spatial structure in populations, multivariate methods can be applied to genetic markers. Geography is explicitly integrated in the spatial principal component analysis (sPCA; Jombart et al. 2008). This approach maximizes variance among genotypes while taking spatial information into account. More precisely, it focuses on the part of the variance that is spatially structured and accounts for the spatial autocorrelation that is associated with sample distribution (Schwartz and McKelvey 2009). To investigate the spatial pattern of genetic variability in the termite population, we used the multilocus genotypes of the individual representative georeferenced termites. The analysis was performed using the adegenet package (Jombart 2008) in R v. 2.7.2 (R Development Core Team, 2011): The 52 *R. grassei* colonies were the analytical units, and each comprised a group of genotypes. After obtaining allele frequencies for each colony from the genotype data, a connection network based on K-nearest-neighbor values (*K* = 10) was applied. First, Moran’s *I* index was calculated to detect any spatial autocorrelation in the allele frequencies. Indeed, we expected that patches of similar allele frequencies could lead to a highly positive *I* because, within a population, the observed allele frequency of a colony should be positively correlated with the allele frequencies of its neighbors. The first sPCA eigenvalues that explained both variance and spatial autocorrelation were retained. The corresponding sPCA principal components (i.e. scores) were geographically mapped to reveal spatial patterns of interest. Second, global and local statistical tests (9999 permutations) were performed to detect global and/or local structuration forces, as per Jombart et al. (2008). Global patterns are present when entities are more genetically similar to their neighbors than expected in populations with random spatial distributions (positive spatial autocorrelation), whereas local patterns occur when entities are more dissimilar than expected (negative spatial autocorrelation) (Jombart et al. 2008). In addition to assessing spatial patterns, we also looked for the alleles that contributed the most to the first and second axes of the sPCA (using the function *loadingplot* in the adegenet package).

### Analyses of genetic structure using Bayesian clustering models

To complement the latter frequency-based methods, we used two Bayesian clustering algorithms, Structure (v. 2.2.3, Pritchard et al. 2000) and Geneland (v. 4.0.4, Guillot et al. 2005), to infer population structure (i.e. the number of clusters, *K*) and to (probabilistically) assign colonies to clusters based on the multilocus genotype data. Both of the algorithms assume that clusters are panmictic units with distinct allele frequencies. In order to circumvent possible problems that might have arisen from the non-independence of genotypes within colonies, all the analyses were performed using data sets in which each colony was represented by a single, randomly chosen individual (see description above).

In the Structure analyses, five runs, each using a different resampled data set, were conducted to look for consistency in the predicted K values. Each run consisted of a burn-in period of length 50,000 followed by 100,000 Markov chain Monte Carlo (MCMC) repetitions with 10 iterations using the admixture model. For the five runs, the optimal value of *K* was calculated using the Δ*K* methods (Evanno et al. 2005) in Structure Harvester v. 0.6 (Earl and Vonholdt 2012).

We used Geneland to infer population structure and genetic boundaries (Guillot et al. 2005, 2009); the Geneland algorithm may better define genetic units because it integrates the spatial coordinates of samples. Colonies were probabilistically assigned to groups using a Bayesian cluster model, which was implemented using MCMC methods (Guillot et al. 2005). *K* was allowed to vary from 1 to 10, and 100,000 MCMC iterations were run using a thinning interval of 100. The Dirichlet model was used as the prior distribution for all the allele frequencies. Initially, we conducted five runs (using five different resampled data sets as above for Structure). Given that the posterior distributions yielded two different modes, we then performed five additional runs to improve our estimation of *K*.

### Standard population genetic analyses of the inferred clusters

Using the online version of Genepop (v. 4.2; Raymond and Rousset 1995), we ran the same standard population genetic analyses as above (i.e. on the full sample) on the results of two Geneland runs that had identified either two or three clusters. We tested for deviations from HWE at each locus and overall; we calculated *F*_IS_ values; and we tested for genotypic linkage disequilibrium. We calculated pairwise *F*_ST_ values between the clusters (as per Weir & Cockerham 1984) using fstat 2.9.3 (Goudet 2001). The significance level for the *F*-statistics was defined using a nonparametric permutation procedure employing 1000 randomizations. Finally, we tested for IBD patterns within all the clusters using the adegenet v.1.4.2 and ade4 v.1.6-2 packages in R 3.0.3.

### Colony breeding systems

Colonies were assigned to one of three family types by comparing their allele and genotype numbers and frequencies with those expected given standard criteria for the respective termite families (Vargo 2003; DeHeer and Vargo 2004). Colonies were categorized as simple families when worker genotypes were consistent with those expected of the direct offspring of a single pair of reproductives. Colonies were considered as extended families when they had no more than four alleles at any one locus and when worker genotypes were inconsistent with those expected of the direct offspring of a single pair of reproductives (*e.g*. more than four genotypes at a locus or three or more homozygous genotypes). A colony was also considered to fall into the extended-family category if its genotype frequencies deviated significantly from those expected in simple-family colonies. Significance was determined by a *G*-test (*P *< 0.05) in which all the loci were combined. Colonies were categorized as mixed families when more than four alleles were found at one or more loci, a pattern that reveals that offspring were produced by more than two unrelated reproductives.

## Results

### Standard population genetic analyses of the full sample

Sequencing of 684 bp of the mtDNA COII locus revealed four polymorphic nucleotide sites that, taken together, resulted in three haplotypes: HT1, HT2, and HT3 (Table S2; accession numbers AY510581, AY510577, and AY510576, respectively; Kutnik et al. 2004). HT1 and HT2 were found throughout the study area and were present in 31 colonies (60%) and 14 colonies (27%), respectively (Fig.1 and Table S1). In contrast, HT3 was found in only 7 colonies in the south (13%).

The mean number of alleles per locus across all colonies was 4.67; it ranged from 2 to 13 (see Table1 for information on the individual loci). There were no significant linkage disequilibria between loci. As a group, the loci deviated significantly from HWE; furthermore, deviations were detected for over 50% of the 20 resampled data sets for most of the loci. This result indicates the presence of homozygote excess. In the two runs that were further analyzed in Geneland, the mean values for overall expected unbiased heterozygosity were 0.505 and 0.483; the mean values for observed heterozygosity were lower, 0.244 and 0.256 (Table1). Global *F*_IS_ was equal to 0.547 and significant for both runs (*P *=* *0.004 and *P *=* *0.008; Table1). The AMOVA revealed that the variance components both among colonies and within individuals were highly significant. About 56% of the microsatellite variance was attributable to divergence among colonies (*P *≤* *0.0001), while the remaining variance (about 44%) could be attributed to variability within individuals (*P *≤* *0.0001; Table S3).

**Table 1:** Summary of the results of the population genetic analyses for the full sample and for the inferred clusters (two or three) obtained from two Geneland runs

Locus name	No. of alleles	*K* = 2 Geneland					*K* = 3 Geneland					
		Full sample			Northern cluster	Southern cluster	Full sample			Northern cluster	Southern cluster	Southwestern cluster
		*H*_O_	*H*_E_ n.b.	*F*_IS_	*F*_IS_	*F*_IS_	*H*_O_	*H*_E_ n.b.	*F*_IS_	*F*_IS_	*F*_IS_	*F*_IS_
*Rf21-1*	13	0.301	0.816	0.647*	0.632*	0.629*	0.281	0.810	0.695*	0.597*	0.866*	0.480
*Rf24-2*	3	0.206	0.370	0.519*	0.408	0.517	0.255	0.302	0.207	0.143	−0.030	0.226
*Rf5-10*	3	0.144	0.391	0.711*	0.658	0.628*	0.180	0.365	0.748*	0.000	0.917*	−0.164
*Rf15-2*	2	0.222	0.273	0.318	0.206	0.000	0.194	0.231	0.268	0.217	0.000	0.000
*Rf6-1*	3	0.305	0.511	0.391*	0.263	0.538	0.284	0.523	0.437*	0.491*	0.242	0.624
*Rs76*	4	0.288	0.665	0.568*	0.580*	0.552*	0.342	0.665	0.615*	0.824*	0.572	0.086
All loci	4.67	0.244	0.505	0.547*	0.455*	0.576*	0.256	0.483	0.547*	0.503*	0.640*	0.283

*H*_O_, observed heterozygosity; *H*_E_ n.b., heterozygosity expected under Hardy–Weinberg equilibrium, corrected for sampling bias. *F*_IS_ was calculated as per Weir & Cockerham (1984) and tested for evidence of a heterozygosity deficiency – the

* indicates that the value was significant (*α *= 0.05) following corrections for multiple comparisons. The last row gives the overall mean number of alleles, mean heterozygosity, and *F*_IS_.

**Figure 2:**
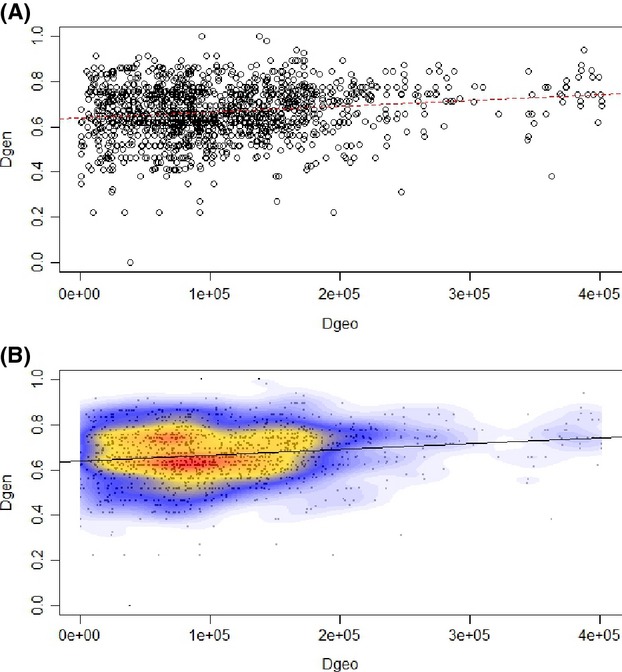
Isolation-by-distance analysis for the 52 *R. grassei* colonies (using one individual per colony). (A) Pairwise Edwards’ distances plotted against Euclidean geographic distances. (B) Local density of points plotted using a two-dimensional kernel density estimation.

There was significant evidence for IBD across the 52 samples (Fig.2A). We can reject the null hypothesis that the genetic and geographical matrices were unrelated (*α *= 0.05). The observed correlation of *r *= 0.162 (*P *=* *0.009; 999 permutations) suggests that the matrices were positively associated. Although the scatterplot (Fig.2B) contains a single main cloud of points, there is also a small group of points on the right side of the graph; it is likely composed of some genetically differentiated colonies that were located rather far from each other.

### Genetic structure revealed by multivariate analyses

**Figure 3:**
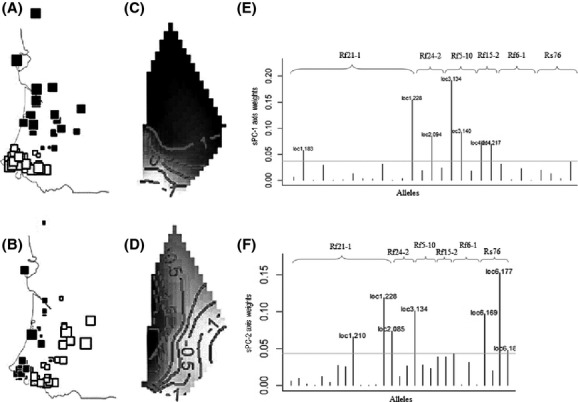
Spatial principal component analysis (sPCA) of the 52 *R. grassei* colonies. (A–B) The colony scores associated with the first two eigenvalues of the sPCA are geographically mapped, a procedure that maximizes both genetic variability and spatial autocorrelation. Each square represents a given colony’s score and is positioned in accordance with the colony’s geographical coordinates. The area of each square is proportional to the absolute value of the colony’s score. The color (black or white) of the square corresponds to the sign of the score (positive or negative, respectively). The size of the square indicates the magnitude of the score on each axis and thus represents each colony’s position relative to the overall genetic structure of the population. (C–D) Contour lines illustrate the orthogonal genetic gradients based on the interpolation of the sPCA scores across the study area. (E–F) Bar graphs show the allelic contributions (i.e. loading weights) to spatial principal components sPC–1 and sPC–2. The height of bars indicates the weight of each allele (based on 6 microsatellite loci) on each sPC axis.

The first two sPCA components, sPC–1 and sPC–2, accounted for 46.7% of the total variance. The eigenvalue for the first sPCA component was strikingly large (*λ*_1_ = 0.17) compared to the others: It explained the high degree of variation in allele frequency. It differentiated all the termite colonies into two groups: a “northern” group and a “southern” group (Fig.3A and C). This pattern was associated with a strong degree of spatial autocorrelation among genotypes (Moran’s index *I *=* *0.618), which revealed that the observed allele frequencies were highly correlated among geographically close colonies. The second sPCA eigenvalue (*λ*_2_ = 0.03) revealed another spatial trend: There was some differentiation among “western” and “eastern” colonies, although the autocorrelation statistic was weaker (*I *=* *0.158; Fig.3B and D). In sPC–1, almost all the genotypes had high scores (larger squares in Fig.3A); in contrast, in sPC–2, some genotypes had scores approaching zero (smaller squares in Fig.3B). This result suggests that spatial structuration in this termite population takes place more along the north–south axis than along the east–west axis. The subsequent sPCA components explained much smaller amounts of the total variance. The global test confirmed the existence of a global pattern within the study area (*P < *0.0001), while the test for local population structure (negative autocorrelation at the local scale) was not significant (*P *=* *0.289). We identified the alleles underlying this pattern. The alleles that contributed the most to sPC–1 were loc1.228 (locus *Rf21-1*) and loc3.134 (locus *Rf5-10*) (Fig.3E). The loc6.177 (locus *Rs76*) and loc1.228 (locus *Rf21-1*) alleles made the largest contributions to sPC-2 (Fig.3F).

### Analyses of genetic structure using Bayesian clustering models

**Figure 4:**
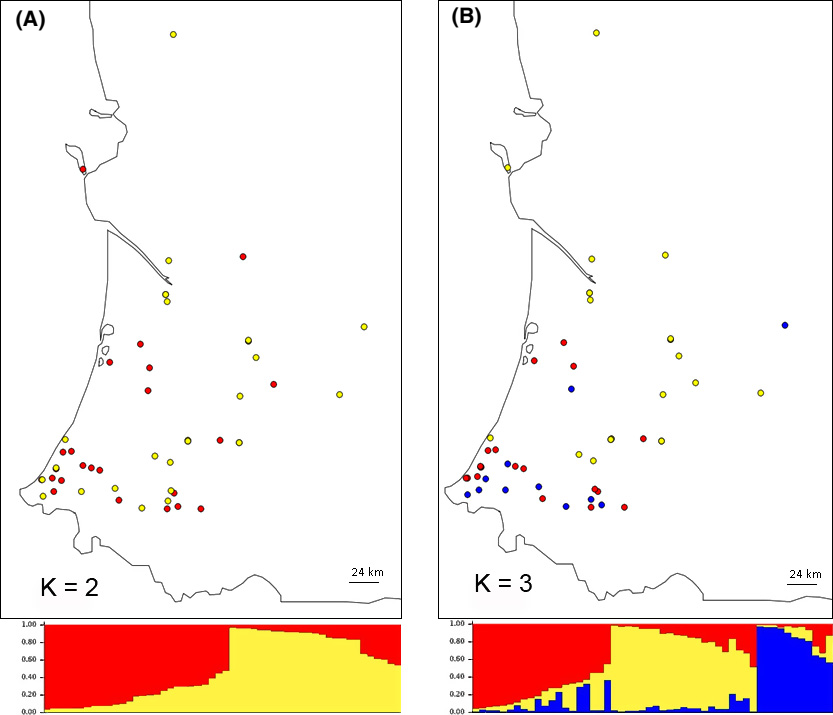
Assignment of the 52 *R. grassei* colonies to clusters identified by five structure runs; a single individual was randomly chosen to represent each colony. Two runs identified two genetic clusters (A), while three runs identified three clusters (B). Each color represents a genetic cluster, and the colors on the graph under the map indicate the likelihood of a colony being assigned to a given cluster. On the map, the dots indicate the sampling sites, and the color of each dot corresponds to the cluster to which the colony was assigned.

The Structure results suggest that our data are best structured by grouping the colonies into two clusters (*K* = 2), according to two of the runs (Fig.4A; Fig. S1: runs #1 and #2), or into three clusters (*K* = 3), according to three of the runs (Fig.4B; Fig. S1: runs #3 to #5). For *K* = 2, 27 colonies were assigned to the first cluster and 25 colonies were assigned to the second cluster (Fig.4A; the colonies forming these clusters are depicted by red and yellow dots, respectively). The two clusters seem to be heterogeneously distributed and thus do not exhibit a particular spatial pattern. In contrast, for *K* = 3, 20 colonies were assigned to the first cluster, 21 colonies to the second cluster, and 11 colonies to the third cluster (Fig.4B; depicted by red, yellow, and blue circles, respectively). There appears to be some overall spatial structure as the yellow colonies have a northern distribution while almost all of the blue colonies have a southern distribution.

**Figure 5:**
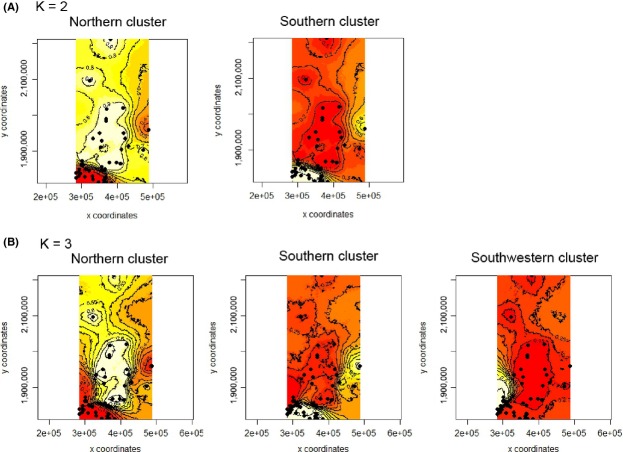
Population structure of the 52 *R. grassei* colonies as estimated by the geneland analyses. Geographical data and the multilocus genotypes of the representative georeferenced individual termites were taken into account, and each colony was randomly resampled to create the data sets used. The maps of the clusters represent the two modal solutions: (A) *K* = 2 (northern and southern clusters) and (B) *K* = 3 (northern, southern, and southwestern clusters). The highest membership values are in light yellow, and the curves indicate spatial changes in assignment values. The plot is based on the highest probability run for a given value of *K*.

In the Geneland analyses, 6 of the 10 runs identified two clusters (*K* = 2): 29 colonies were assigned to the first cluster and 23 colonies were assigned to the second cluster (numbers of colonies were averaged across the 6 runs). The maps of estimated cluster membership (based on the posterior modes) suggest that the first, main cluster has a more diffuse, northern distribution and that the second cluster has a more southern distribution (with the exception of colony #52). There is a distinct border between the clusters (below colonies #42 and #43; Figs.1, 5A). The four other runs identified three clusters (*K* = 3) and proposed a different version of this general pattern. On the map, it can be seen that the northern cluster (composed of 26 colonies) remains, but that there are two distinct clusters in the south (Fig.5B). The first (17 colonies) is situated in the southern part of the study area, and the second (9 colonies) is located more to the southwest. For both *K* = 2 and *K* = 3, numbers of colonies were averaged across runs.

### Standard population genetic analyses of the inferred clusters

We conducted standard population genetic analyses on the results of two Geneland runs that had identified either two or three clusters. There were no significant linkage disequilibria between loci in any of the clusters. For *K* = 2, *F*_IS_ equaled 0.455 and 0.576 for the northern and southern clusters, respectively; there were also significant deviations from HWE (*P *=* *0.0042 for both clusters) (Table1). In both the northern and southern clusters, this result was attributable to two loci, *Rf21-1* and *Rs76*; *Rf5-10* also had a significant influence in the southern cluster (Table1). For *K* = 2, the pairwise *F*_ST_ was significant (0.1385; *P *=* *0.04). For *K* = 3, the northern and southern clusters had significant *F*_IS_ values (0.503 and 0.640, respectively), which were attributable to the following loci: *Rf21-1*, *Rf6-1*, and *Rs76* for the northern cluster and *Rf21-1* and *Rf5-10* for the southern cluster (Table1). In contrast, the southwestern cluster did not have a significant *F*_IS_ value (0.283). The pairwise *F*_ST_ values for the northern and southern clusters and for the northern and southwestern clusters were significant (0.1467, *P *=* *0.0167 and 0.1149, *P *=* *0.0167, respectively). In contrast, no significant difference was detected between the southern and southwestern clusters (0.1029, *P *=* *0.0667). There were no significant IBD patterns within any of the clusters (*K* = 2: northern cluster: *r* = 0.118, *P *=* *0.117 and southern cluster: *r* = −0.053, *P *=* *0.671; *K* = 3: northern cluster: *r *= 0.073, *P *=* *0.247, southern cluster: *r* = −0.092, *P *=* *0.746, and southwestern cluster: *r* = 0.016, *P *=* *0.44).

### Colony breeding systems

We identified 20 simple-family colonies (38.5%) and 32 extended-family colonies (61.5%) (Table S1) using a standard system of colony classification based on genotype distributions at all microsatellite loci (Vargo 2003). No mixed-family colonies were found in our study. Using the Geneland clustering results, we estimated the percentages of each family type in the different clusters. For *K* = 2, in the northern cluster, 33% and 67% of the colonies were simple family and extended family, respectively; in the southern cluster, 40% and 60% of the colonies were simple family and extended family, respectively. Similarly, for *K* = 3, in the northern, southern, and southwestern clusters, 37.5%, 42%, and 22% of colonies were simple family, respectively, while 62.5%, 58%, and 78% were extended family, respectively.

## Discussion

### Mitochondrial genetic diversity

*Reticulitermes grassei* colonies in our study area in southwestern France demonstrated limited mitochondrial DNA diversity: Only three haplotypes were observed. These haplotypes were previously described in a study that examined *R. grassei* and *R. banyulensis* phylogeography at other locations in southwestern France and on the Iberian Peninsula (Kutnik et al. 2004). The authors found HT1 in four French colonies (located near Chatellerault, Parentis, Bouglon, and Caudecoste) and one Spanish colony (Arborteretza) and discovered HT3 in one French colony (Bayonne). In contrast, HT2 was detected in a single Spanish colony (Arevalo) but was absent from French colonies. Genetic diversity was greatest in Spain and northern Portugal, where 14 other COII haplotypes were found across the 20 colonies studied (Kutnik et al. 2004). Furthermore, 20 COII haplotypes (527 bp) were observed along a 640-km north–south transect in Portugal (Nobre et al. 2006), and a recent study detected more than 22 COI-COII haplotypes on the Iberian Peninsula and Morocco (Lefebvre et al. submitted). Both of these results emphasize that diversity was low in our study area. They also suggest that colonies dispersed from Spain and colonized southern France over the course of several postglacial events (Lefebvre et al. submitted); support for this hypothesis comes from several markers (cuticular hydrocarbons, nuclear DNA sequences, and microsatellites). The geographical distribution of the three haplotypes in southern France, and particularly the limited presence of HT3 in the south, prompted our investigation of the population genetic structure of this termite species. While the mitochondrial DNA data have clarified the evolutionary history of this population, the genotype data and population genetic analyses have revealed how contemporary processes, such as dispersal and gene flow, are operating in colonies across all of southwestern France.

### Quantifying spatial genetic structure

The analysis of large-scale population genetic structure revealed an overall deviation from HWE in the study area. The significant level of homozygote excess that we observed (global *F*_IS_ = 0.547) may be attributable to a Wahlund effect. In social insects, homozygote excess can also be a consequence of inbreeding among related reproductives within colonies (Dronnet et al. 2005; Perdereau et al. 2010). Therefore, we first addressed the possibility that the deviation from HWE was due to the presence of discrete genetic subpopulations within the study area. On the whole, levels of differentiation were high: 56% of the genetic variance was due to differences among colonies (AMOVA, Table S3). We also found evidence that genetic differentiation among individuals (the representatives from the 52 study colonies) increased significantly with geographical distance, which could account for the spatial population genetic structure observed. However, IBD patterns depend on spatial scale and do not necessarily imply homogeneous gene flow across space (Slatkin 1993; Rousset 1997). Some authors have already empirically shown that significant IBD patterns may exist even if barriers to gene flow are present, because populations or clusters are genetically differentiated (*e.g*. Garnier et al. 2004). While our results seem to suggest the presence of IBD, we did not observe a continuous cline of genetic differentiation. Instead, our data revealed a discontinuity in the form of a patch (Fig.2B). This heterogeneous distribution can be attributed to the genetically differentiated colonies in the south, an interpretation that is supported by the other, complementary analyses we conducted.

As it was crucial to be able to identify whether the population was structured by barriers to gene flow, we analyzed allele frequencies using multivariate methods to explore spatial patterns of genetic differentiation within the population. The spatial principal component analysis (sPCA) uses allele frequency data to calculate spatial autocorrelation scores and thus tests for spatial patterns of genetic variability (Jombart et al. 2008). We found significant evidence that the positive autocorrelation we found (i.e. observed allele frequencies were highly correlated among geographically close colonies) influenced overall population structure within the study area (global test, *P *<* *0.0001). First, it is clear from the pattern that the colonies were separated into a “northern” and a “southern” group, and the boundary between the two groups is demarcated by the near-zero sPCA scores for colonies #13, #40, #41, #42, and #43 (Figs.1, 3A and C). Second, we also detected a weaker east–west differentiation (Fig.3B and D). To explore the underlying genetics, we visually identified the alleles that contributed the most to population structure as captured by sPC-1 and sPC-2. Interestingly, between-group differences can be explained by the possession of a few alleles at some loci that are not necessarily highly polymorphic.

These frequency-based analyses were then complemented by model-based clustering methods, which allowed us to (1) assign colonies to clusters while accounting for individual genotypes and (2) detect genetic discontinuities. It is important to note that, while our data suggested the presence of an IBD pattern, the results of the clustering analyses identified two or three clusters, depending on the resampled data set. Although the structure analyses did not reveal any sharp discontinuities, a spatial pattern was nonetheless present; in particular, two distinct clusters were found to exist in the south. The geneland analyses, which incorporated spatial information on colony location, also identified two or three clusters, which indicates that there is a clear spatial structure within the study area. Again, the number of clusters was run dependent. For *K* = 3, we found that the northern and southern clusters were highly differentiated, as were the northern and southwestern clusters. However, the southern and southwestern clusters were not, suggesting the occurrence of some gene flow between them.

The next step was to look for landscape features that could account for the observed genetic patterns and discontinuities (Manel et al. 2003). The main potential physical barriers to termite dispersal are the area’s steep topography and the presence of several moderately sized rivers that flow from east to west, from the Pyrenees Mountains to the sea (*e.g*., the Adour River). In general, *Reti culitermes* winged primary reproductives naturally disperse only short distances (reviewed in Vargo and Husseneder 2011); for example, *R. flavipes* does not disperse farther than 460 m in its native range (Shelton et al. 2006). We hypothesize that the limited dispersal ability of *Reticulitermes* species could explain the observed genetic structure of *R. grassei*; habitat composition may also be involved. In addition to these natural factors, the transport of infested anthropogenic materials may also play an important role in maintaining some gene flow among populations. We found no evidence of IBD patterns within clusters, but there was a significant heterozygosity deficit in the northern and southern clusters for both *K* = 2 and *K* = 3: *F*_IS_ values were high. These deficits could be due either to some sort of hidden population structure (i.e. a Wahlund effect) or inbreeding among related reproductives within colonies; the latter possibility will be discussed below. We did not detect a deficit in the southwestern cluster, possibly because its smaller sample size led to reduced statistical power.

### Influence of termite breeding systems on population structure

Colony breeding systems directly influence both the level of inbreeding among reproductives and the modes of dispersal, which in turn affect colony genotypic diversity and spatial distribution (reviewed by Vargo and Husseneder 2009, 2011). Simple-family colonies are founded by winged primary reproductives (a queen and an unrelated king), generally following an annual swarming event (most of the time, dispersal is enhanced by the presence of wind); this process results in high levels of gene flow (Thorne et al. 1999). Offspring in mixed-family colonies are produced by more than two unrelated primary reproductives; such colonies may result from colony fusion, as is the case in *R. flavipes* (DeHeer and Vargo 2004; Perdereau et al. 2010, 2015). Finally, extended-family colonies are created when secondary neotenic reproductives that descend from the founding primary reproductives supplement breeding by the latter or replace them entirely, thus increasing the level of inbreeding. Such colonies commonly disperse by budding, whereby workers and secondary reproductives initiate new colonies in close proximity to their natal nests (Thorne et al. 1999). As such colonies become larger over time, they may also produce some winged primary reproductives, who may enhance gene flow although they themselves are inbred. Most of the time, long-range dispersal occurs when colonies are fragmented via human-mediated transport. In this study, we observed a large percentage of extended-family colonies (61.5%). The simultaneous occurrence of both simple- and extended-family colonies has already been documented in previous studies (DeHeer et al. 2005; Nobre et al. 2008; Perdereau et al. 2010; Vargo et al. 2013) that intensively sampled eight populations of *R. grassei* across entire forests; extensive intercolonial variation in breeding systems was observed in France, Portugal, and Spain. Recently, Vargo et al. (2013) examined how geographical variation in *R. grassei* colony breeding systems across southern Spain, Portugal, and southwestern France may be explained by environmental factors. Wood substrate availability and soil composition seem to be important predictors of inbreeding in this species (Vargo et al. 2013). The authors found a significant correlation between latitude and the relative level of inbreeding (individual level versus population level) in *R. grassei*: Inbreeding increased from south to north. They also detected a trend in breeding systems, from simple families in the southern parts of the species’ range to extended families in the northern parts of the range. Interestingly, our research corroborates this finding as extended families were prevalent in our study area, which lies at the northern limit of *R. grassei*’s natural distribution in France. We suggest that the latitudinal range explored here is not large enough to detect clinal variation in colony breeding systems.

## Conclusions

Our findings have important implications for our understanding of the role different factors (*e.g*., the local environment and species biology) play in determining population genetic structure in *Reticulitermes*. Our study has demonstrated that using complementary analyses is especially crucial when dealing with social species in which both spatial and social constraints interact to shape the distribution of intraspecific diversity. The next step is to conduct a study at the scale of the Landes’ forest, whose recent history (it was planted at the beginning of the 1800s) and its particularly large area (2.47 million acres covered mainly with the pine species *Pinus pinaster*) have probably helped the termite move northward and have influenced its population structure; human activities (e.g. the transport of infested trees or anthropogenic materials) may also have had an effect.

## Data Accessibility

COII sequences: GenBank accession numbers AY510576, AY510577, and AY510581.

Microsatellite genotypes data: http://datadryad.org/resource/doi:10.5061/dryad.vq0c8/1.

## Supporting Information

**Table S1.** Locations and genetic characteristics of the 52 *Reticulitermes grassei* colonies sampled in southwestern France. **Table S2.** DNA sequence variation among the three COII mitochondrial haplotypes. **Table S3.** Analysis of molecular variance (AMOVA) incorporating three hierarchical levels: among colonies, among individuals within colonies, and within individuals. **Figure S1.** DeltaK plot for determining *K*, the number of clusters (Evanno et al. 2005) formed by the 52 *Reticulitermes grassei* study colonies according to the Structure Harvester analyses; five replicate runs were conducted for which each colony was randomly resampled. The best-fit number of clusters was two for two of the runs and three for the other three runs.
