# Supplementary material for: Spatial structuring of the population genetics of a European subterranean termite species
Source: Ecol Evol. 2015 Jul 8;5(15):3090–102. doi: 10.1002/ece3.1566 (PMC4559052; doi:10.1002/ece3.1566)
Supplement: Supplementary file 1 [file ece30005-3090-sd1.doc]

**Additional supporting information – *Ecology and Evolution***

**Spatial structuring of the population genetics of a European subterranean termite species**

S. Bankhead-Dronnet, E. Perdereau, M. Kutnik, S. Dupont and A.-G. Bagnères

**Table S1.**  Locations and genetic characteristics of the 52 *Reticulitermes grassei* colonies sampled in southwestern France; a COII mtDNA fragment and 6 microsatellite loci were characterized. For the mtDNA, the three haplotypes (HT) are mentioned HT1, HT2 or HT3. For the microsatellite loci, the following descriptors are provided: N = sample size; N*_PM_* = number of polymorphic microsatellites; mean observed (H*o*) and unbiased expected (H*e*) heterozygosity. Colonies were characterized by their breeding system: simple or extended families.

| **Colony** | **Location**  **(county)** | **Latitude** | **Longitude** | **mtDNA HT** | **N** | **N*_PM_*** | **H*o*** | **H*e*** | **Breeding systems** |
| --- | --- | --- | --- | --- | --- | --- | --- | --- | --- |
| **1** | Peyrehorade (40) | 43.548333 | -1.111111 | HT1 | 7 | 1 | 0.12 | 0.08 | simple |
| **2** | Bayonne (64) | 43.491389 | -1.477222 | HT3 | 10 | 3 | 0.23 | 0.23 | extended |
| **3** | Biarritz (64) | 43.478056 | -1.553333 | HT1 | 10 | 5 | 0.45 | 0.42 | extended |
| **4** | Biarritz (64) | 43.480556 | -1.562778 | HT3 | 10 | 5 | 0.38 | 0.39 | extended |
| **5** | St-Pée sur Nivelle (64) | 43.355556 | -1.550000 | HT2 | 10 | 1 | 0.03 | 0.08 | simple |
| **6** | Ayherre (64) | 43.391389 | -1.253611 | HT3 | 10 | 2 | 0.13 | 0.17 | extended |
| **7** | Rontignon (64) | 43.261389 | -0.329722 | HT2 | 10 | 3 | 0.23 | 0.21 | extended |
| **8** | Domezain (64) | 43.325556 | -0.965556 | HT2 | 10 | 2 | 0.07 | 0.13 | extended |
| **9** | Labastide-Cézéracq (64) | 43.378056 | -0.536389 | HT2 | 10 | 3 | 0.22 | 0.16 | extended |
| **10** | Cardesse (64) | 43.261111 | -0.588056 | HT1 | 10 | 2 | 0.10 | 0.11 | extended |
| **11** | Ondres (40) | 43.561111 | -1.447778 | HT1 | 10 | 3 | 0.30 | 0.28 | extended |
| **12** | Ondres (40) | 43.562500 | -1.449444 | HT1 | 10 | 4 | 0.45 | 0.34 | simple |
| **13** | Ondres (40) | 43.566667 | -1.450278 | HT1 | 10 | 4 | 0.22 | 0.28 | simple |
| **14** | Tosse (40) | 43.691944 | -1.330278 | HT3 | 10 | 6 | 0.47 | 0.47 | extended |
| **15** | Grenade-s/Adour (40) | 43.772778 | -0.428333 | HT1 | 10 | 5 | 0.39 | 0.39 | simple |
| **16** | Port-de-Lanne (40) | 43.565000 | -1.177500 | HT1 | 10 | 4 | 0.35 | 0.35 | simple |
| **17** | Le Houga (47) | 43.774444 | -0.180556 | HT1 | 10 | 5 | 0.25 | 0.36 | simple |
| **18** | Sabres (40) | 44.148333 | -0.740000 | HT1 | 10 | 2 | 0.15 | 0.14 | extended |
| **19** | La Roche Chalais (24) | 45.152222 | -0.007500 | HT1 | 10 | 4 | 0.26 | 0.32 | simple |
| **20** | Cars (33) | 45.119444 | -0.580556 | HT2 | 10 | 3 | 0.17 | 0.18 | extended |
| **21** | Talence (33) | 44.812500 | -0.590833 | HT1 | 10 | 4 | 0.33 | 0.28 | simple |
| **22** | Le Bouscat (33) | 44.865278 | -0.600000 | HT1 | 10 | 4 | 0.33 | 0.34 | extended |
| **23** | Le Bouscat (33) | 44.868056 | -0.601389 | HT1 | 10 | 5 | 0.35 | 0.39 | extended |
| **24** | Châtellerault (86) | 46.821111 | -0.544444 | HT1 | 10 | 4 | 0.14 | 0.22 | extended |
| **25** | Caudecoste (47) | 44.115833 | 0.737500 | HT1 | 10 | 4 | 0.24 | 0.23 | simple |
| **26** | Bouglon (47) | 44.392500 | 0.097222 | HT1 | 10 | 6 | 0.36 | 0.40 | extended |
| **27** | Nogaro (32) | 43.756944 | -0.035278 | HT1 | 10 | 4 | 0.26 | 0.35 | extended |
| **28** | Nogaro (32) | 43.758333 | -0.037500 | HT1 | 9 | 6 | 0.44 | 0.49 | extended |
| **29** | Meilhan s/Garonne (47) | 44.519444 | 0.033611 | HT1 | 9 | 3 | 0.08 | 0.08 | extended |
| **30** | Meilhan s/Garonne 47) | 44.520833 | 0.035000 | HT1 | 10 | 0 | 0.00 | 0.00 | extended |
| **31** | Meilhan s/ Garonne (47) | 44.523056 | 0.035556 | HT1 | 9 | 3 | 0.26 | 0.23 | simple |
| **32** | Artix (64) | 43.395278 | -0.560000 | HT2 | 9 | 5 | 0.32 | 0.28 | simple |
| **33** | Belin-Beliet (33) | 44.496389 | -0.798333 | HT2 | 10 | 6 | 0.33 | 0.34 | extended |
| **34** | Gatseau (17) | 45.806944 | -1.238333 | HT2 | 10 | 4 | 0.40 | 0.35 | extended |
| **35** | Haut-Richet (40) | 44.318611 | -0.725278 | HT1 | 10 | 5 | 0.35 | 0.35 | extended |
| **36** | Seignosse (40) | 43.686389 | -1.392222 | HT1 | 10 | 3 | 0.30 | 0.28 | extended |
| **37** | Vieux-Boucau (40) | 43.782222 | -1.375278 | HT3 | 9 | 5 | 0.52 | 0.42 | simple |
| **38** | Lubbon (40) | 44.103889 | -0.027222 | HT2 | 10 | 2 | 0.15 | 0.10 | simple |
| **39** | Petit Bôo (40) | 44.357500 | -1.033056 | HT1 | 10 | 4 | 0.36 | 0.26 | extended |
| **40** | Grenade s/Adour (40) | 43.766111 | -0.431389 | HT1 | 10 | 3 | 0.28 | 0.25 | extended |
| **41** | Grenade s/Adour (40) | 43.767222 | -0.432500 | HT1 | 10 | 2 | 0.18 | 0.13 | simple |
| **42** | StCricq Chaliesse (40) | 43.653889 | -0.684444 | HT1 | 10 | 2 | 0.12 | 0.13 | extended |
| **43** | Morganx (40) | 43.606667 | -0.569444 | HT1 | 10 | 4 | 0.25 | 0.25 | extended |
| **44** | Campet forest (47) | 44.192222 | 0.228889 | HT2 | 10 | 5 | 0.35 | 0.29 | extended |
| **45** | Ustaritz (64) | 43.391111 | -1.463889 | HT2 | 10 | 2 | 0.12 | 0.09 | extended |
| **46** | Lacommande (64) | 43.275278 | -0.506944 | HT1 | 10 | 1 | 0.04 | 0.08 | simple |
| **47** | Lay (64) | 43.263889 | -0.784722 | HT3 | 10 | 2 | 0.18 | 0.19 | extended |
| **48** | Mouguerre (64) | 43.470000 | -1.409167 | HT3 | 10 | 3 | 0.11 | 0.15 | extended |
| **49** | Abitain (64) | 43.413333 | -0.993611 | HT2 | 10 | 3 | 0.21 | 0.19 | simple |
| **50** | Monnein (64) | 43.321389 | -0.586667 | HT1 | 10 | 1 | 0.03 | 0.03 | simple |
| **51** | St-Martin de Hinx (40) | 43.584444 | -1.239167 | HT2 | 10 | 2 | 0.13 | 0.11 | simple |
| **52** | Saint-Chaliès (47) | 44.624167 | 0.927222 | HT2 | 10 | 3 | 0.15 | 0.16 | extended |

**Table S2.** DNA sequence variation among the three COII mitochondrial haplotypes observed in *Reticulitermes grassei* colonies in southwestern France. The number of colonies containing each haplotype is indicated.

| *Haplotype* | |  | *Base position in sequence* | | | |
| --- | --- | --- | --- | --- | --- | --- |
| *Code* | *GenBank* | *No.* | *42* | *198* | *204* | *369* |
| HT1 | AY510581 | 31 | C | A | C | T |
| HT2 | AY510577 | 14 | T | G | T | T |
| HT3 | AY510576 | 7 | − | − | − | C |

**Table S3.** Analysis of molecular variance (AMOVA) incorporating three hierarchical levels: among colonies, among individuals within colonies, and within individuals.

| Among colonies | | |  | Among individuals within colonies | | | | Within indiv*i*duals | | |
| --- | --- | --- | --- | --- | --- | --- | --- | --- | --- | --- |
| V*_a_* | Percentage | *P* |  | V*_b_* | Percentage | *P* |  | V*_c_* | Percentage | *P* |
| 0.8068 | 56.15 | <0.0000 |  | -0.0074 | -0.51 | 0.6813 |  | 0.6375 | 44.36 | <0.0000 |

**Figure S1.** DeltaK plot for determining K, the number of clusters (Evanno *et al.* 2005) formed by the 52 *Reticulitermes grassei* study colonies according to the Structure Harvester analyses; five replicate runs were conducted for which each colony was randomly resampled. The best-fit number of clusters was two for two of the runs and three for the other three runs.

**
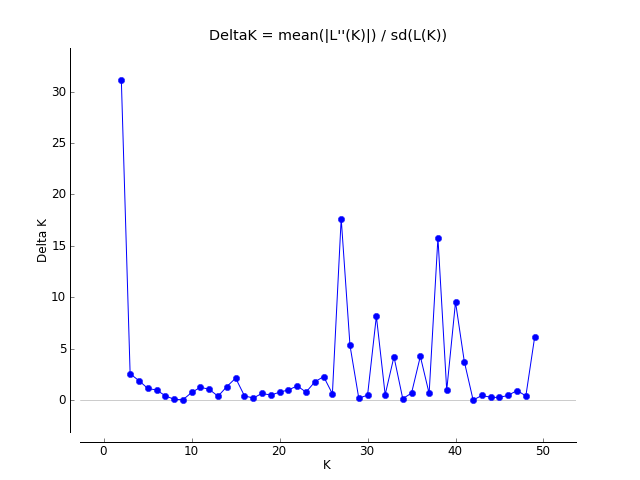

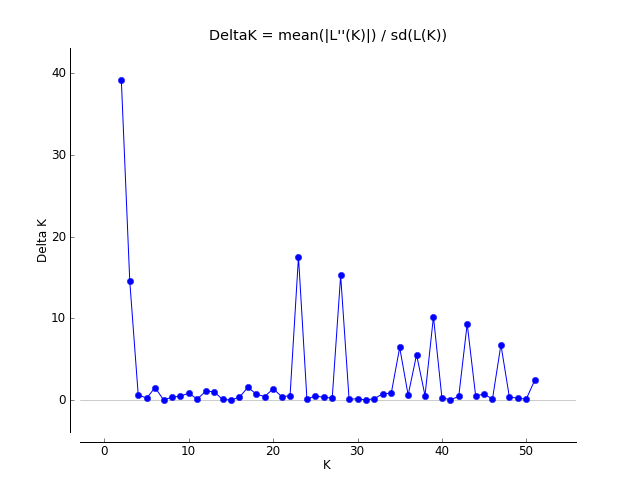
**

Run 1

Run 2

**
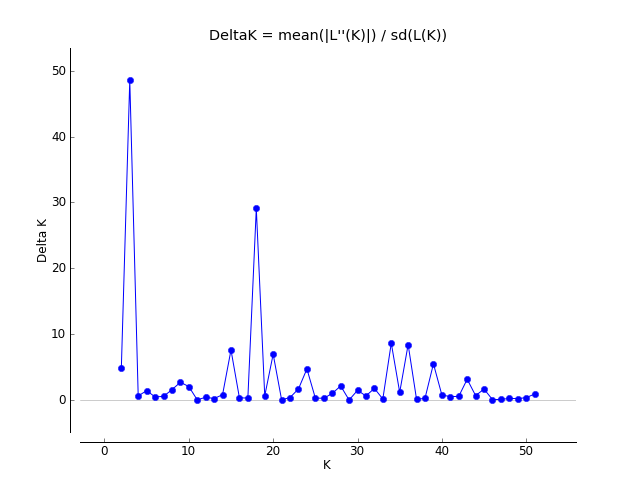

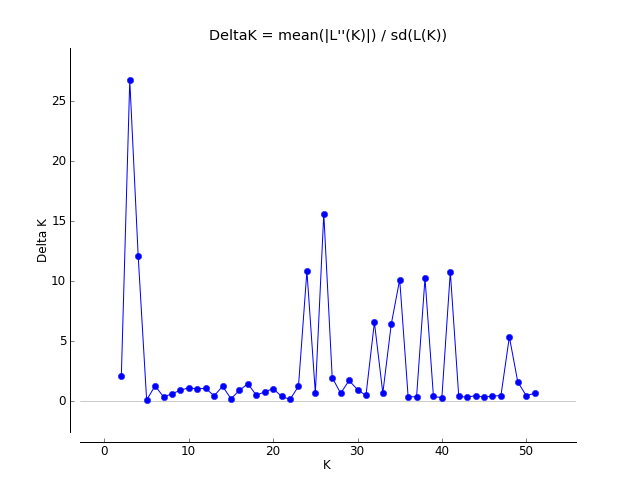
**

Run 4

Run 3

**
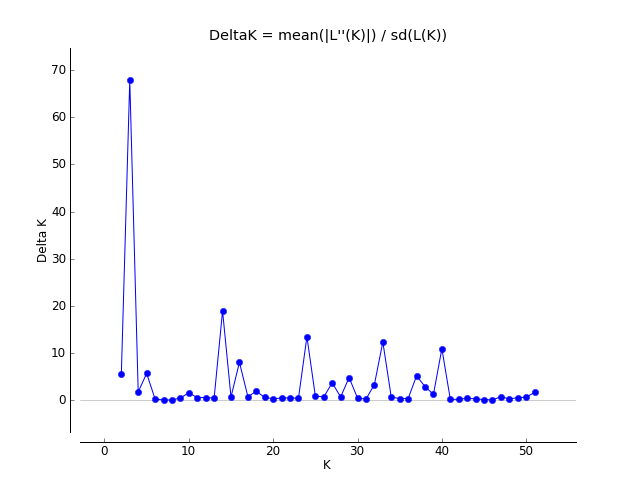
**

Run 5
